# ERK signaling for cell migration and invasion

**DOI:** 10.3389/fmolb.2022.998475

**Published:** 2022-10-03

**Authors:** Shiela C. Samson, Akib M. Khan, Michelle C. Mendoza

**Affiliations:** ^1^ Department of Oncological Sciences, University of Utah, Salt Lake City, UT, United States; ^2^ Huntsman Cancer Institute, University of Utah, Salt Lake City, UT, United States

**Keywords:** RAS, ERK, pulse, actin, adhesion

## Abstract

The RAS - Extracellular signal-regulated kinase (RAS-ERK) pathway plays a conserved role in promoting cell migration and invasion. Growth factors, adhesion, and oncogenes activate ERK. While historically studied with respect to its control of cell proliferation and differentiation, the signaling pattern and effectors specific for cell migration are now coming to light. New advances in pathway probes have revealed how steady-state ERK activity fluctuates within individual cells and propagates to neighboring cells. We review new findings on the different modes of ERK pathway stimulation and how an increased baseline level of activity promotes single cell and collective migration and invasion. We discuss how ERK drives actin polymerization and adhesion turnover for edge protrusion and how cell contraction stimulates cell movement and ERK activity waves in epithelial sheets. With the steady development of new biosensors for monitoring spatial and temporal ERK activity, determining how cells individually interpret the multiple *in vivo* signals to ERK is within reach.

## Introduction

The Extracellular signal-Regulated Kinase (ERK) is a Mitogen-Activated Protein Kinase (MAP kinase) that promotes migration across a broad spectrum of cell types. ERK is the major effector of the small GTPase RAS. Decades of research have identified a large repertoire of ERK effectors involved in cell proliferation, differentiation, and migration, as well as other cellular processes (Mendoza et al., 2011a; Lavoie et al., 2020). Initial work on the signaling for proliferation versus differentiation found that ERK signal intensity, timing, and scaffolds dictate the phenotypic response (Murphy and Blenis, 2006; McKay and Morrison, 2007; Casar and Crespo, 2016; Lavoie et al., 2020). While a seminal study by the Eisuke Nishida lab in the early 2000s suggested that ERK activity traveled through epithelial sheets to promote migration (Matsubayashi et al., 2004), the signaling pattern that controlled migration remained elusive. Recent advances in biosensors and optogenetics (Hirata and Kiyokawa, 2019) are now uncovering the details of the signal transmission that promotes cell migration and invasion.

### ERK pathway signaling

The prototypical RAS→RAF→MEK→ERK signaling pathway has been covered in reviews on RAS-ERK biochemistry and hyperactivation in cancer (Roberts and Der, 2007; Mendoza et al., 2011a; Lavoie et al., 2020). Briefly, Epidermal Growth Factor Receptor (EGFR) is the canonical activator of RAS-ERK signaling (Roberts and Der, 2007; Mendoza et al., 2011a; Lavoie et al., 2020). In growth factor-mediated ERK activation, receptor tyrosine kinases at the cell surface signal to ERK by recruiting the RAS GTPase Exchange Factor (GEF) SOS to the membrane (Mendoza et al., 2011a). SOS activates RAS, which then recruits the kinase RAF. Membrane recruitment activates RAF, which phosphorylates and activates MEK, which in turn phosphorylates and activates ERK (Mendoza et al., 2011a). Chemokines signal through G-Protein-Coupled Receptors, which activate RAS GEFs and RAF through intermediate signaling kinases: Protein Kinase A, Protein Kinase C, and SRC (Mendoza et al., 2011a). ERK is also activated by the binding of integrin receptors to extracellular matrix (ECM), such as fibronectin (SenGupta et al., 2021). When the growth factor concentration is low or the cells are in soft, more physiological environments, fibronectin or integrin clustering are also needed for ERK activation (Slack-Davis et al., 2003; Farahani et al., 2021). Integrin engagement activates EGFR clustering and SRC for RAS activation (Farahani et al., 2021). Integrins also signal to ERK through the recruitment of p21 Activated Kinase (PAK) (Lavoie et al., 2020). PAK phosphorylates and activates RAF and MEK within a scaffold complex in cell-substrate adhesions (Slack-Davis et al., 2003; Nayal et al., 2006; Stockton et al., 2007). Upon activation, ERK acts on nearby substrates, and also translocates to the nucleus where it acts on transcription factors (Lavoie et al., 2020). ERK activity at the cell edge peaks around 5 min after stimulation, while nuclear translocation peaks within 15–30 min and is retained for several hours (Lidke et al., 2010; Mendoza et al., 2011b).

### Cell migration

Cells typically migrate by moving through cycles of edge protrusion, adhesion, and cell body contraction, called the mesenchymal migration mode (SenGupta et al., 2021). Edge protrusion is driven by actin polymerization-mediated pushing force and adhesion-generated traction force, in which cells adhere to the ECM. Then, adhesions in the back of the cell disassemble and myosin II mediates contraction of the cell body for forward translocation (Lauffenburger and Horwitz, 1996). Cells invade (migrate in 3D) along tracks of ECM, neurons, and vessels in the canonical mesenchymal fashion. Tissues and most cancer cells migrate as collectives in sheets or chains, in which cell-cell adhesions are maintained (Friedl and Wolf, 2010; Petrie and Yamada, 2016; Paul et al., 2017). Leader cells at the edge of the sheet or chain exhibit edge protrusion and contraction dynamics similar to cells undergoing single cell migration (Omelchenko et al., 2003; Friedl and Wolf, 2010; Reffay et al., 2014; SenGupta et al., 2021).

Leading edge protrusion is often used as an indicator of cell migration, as it drives the direction and efficiency of movement. Protrusions develop with lamellipodia and lamella structures. At the periphery, the lamellipodia is driven by rapidly assembling, dense actin and nascent adhesions (Watanabe and Mitchison, 2002; Ponti et al., 2004). Here, the actin nucleator ARP2/3 assembles branched actin against the plasma membrane, which pushes the membrane forward (Millius et al., 2012; Mendoza et al., 2015). The small GTPases RAC and CDC42 activate the Wave Regulatory Complex (WRC) and WASp at the cell edge, which recruit and activate ARP2/3 (Stradal and Scita, 2006; Weiner et al., 2007; Chen et al., 2010). As the protrusion progresses, the membrane is stretched and membrane tension increases, which pushes back on the actin filaments and induces their retrograde flow (Raucher and Sheetz, 2000; Ponti et al., 2004; Ji et al., 2008).

Nascent adhesions form as small, punctate structures that rapidly assemble and disassemble (Choi et al., 2008). Integrin binding to ECM recruits focal adhesion proteins that physically connect the integrin-ECM complex to the actin undergoing retrograde flow (Gardel et al., 2010; Parsons et al., 2010). The actin-integrin connection transmits traction (Parsons et al., 2010; Case and Waterman, 2015). Tension across adhesions recruits and activates the scaffold Paxillin and Focal Adhesion Kinase (FAK) (Zhang et al., 2008; Pasapera et al., 2010; Austen et al., 2015), which in turn recruit proteins for adhesion assembly (Laukaitis et al., 2001; Choi et al., 2008; Pasapera et al., 2010). Nascent adhesions undergo a brief stabilization phase and then turn over in response to FAK de-phosphorylation (Nayal et al., 2006; Kuo et al., 2011). As the leading edge moves forward, the remaining older adhesions experience more tension as they resist rearward actin flow and myosin II motors contract actin bundles (Giannone et al., 2007; Anderson et al., 2008; Choi et al., 2008). As a result, the adhesions elongate and strengthen into large, stable focal adhesions resident in the lamella and cell body (Ponti et al., 2004; Giannone et al., 2007; Choi et al., 2008). Myosin II controls cell body contraction by pulling on actin fibers coupled to stable adhesions and the cell membrane (Vicente-Manzanares et al., 2009; Fehon et al., 2010; Parsons et al., 2010). The small GTPase RHO and RHO kinase (ROCK) induce myosin activity (Julian and Olson, 2014; Sadok and Marshall, 2014). While traction force is needed for and promotes cell migration, excessive myosin II activity can induce leading edge retraction and overall cell contraction (Giannone et al., 2004; Ponti et al., 2004; Gupton et al., 2005; Gupton and Waterman-Storer, 2006; Giannone et al., 2007; Ji et al., 2008).

### ERK signaling patterns for migration and invasion

Experiments probing ERK activity in cell migration and invasion have employed global growth factor treatment, activating mutation, and/or the generation of a wound to activate ERK. Global growth factor treatment induces brief periods of directional migration interrupted with pauses and re-direction, called random-walk migration (Wu et al., 2014). This unlocalized signal for ERK activation models the environment encountered by cancer cells spreading into a tissue. The periods of directional migration involve the same protrusion, adhesion, and contraction steps undertaken by cells moving up chemical gradients, which occurs during development and immune and cancer cell homing to specific tissues. In epithelial sheets, the protrusion of leader cells into a new wound initiates directional migration into the wound.

Early experiments suggested that lower concentrations of growth factor signal for cell migration. Many cell types undergo edge protrusion dynamics, random-walk migration, and collective migration into a wound when grown in medium with 5–10% fetal bovine serum, which contains growth factors in the 10–100 pg/ml range (Matsubayashi et al., 2004; Mendoza et al., 2011b; Mendoza et al., 2015; Aoki et al., 2017; Samson et al., 2019). Individual cells undergoing random walks or traveling within collectively migrating sheets will pause and divide. However, a study in NIH3T3 cells suggested that specific ERK signaling parameters might induce cell migration versus proliferation. Low concentrations of Platelet-Derived Growth Factor (PDGF, 1–5 ng/ml) best induced cell migration into a wound, while higher concentrations (≥5 ng/ml) induced cell proliferation (De Donatis et al., 2008). The lower PDGF concentrations resulted in lower levels of phospho- (activated) ERK in the cell population (De Donatis et al., 2008), but the signaling dynamics of individual cells was untested.

The development and study of ERK biosensors and optogenetics tools have begun to reveal the specific ERK dynamics that promote single and collective modes of migration and invasion. In the presence of growth factors, cells undergo pulses of ERK activity with frequency and duration dependent on the specific growth factor, concentration, and cell density (Albeck et al., 2013; Aoki et al., 2013; Hiratsuka et al., 2015). Imaging of a FRET-based cytoplasm-localized ERK biosensor (EKAR-EV (Komatsu et al., 2011)) in mammary epithelial cells showed that low concentrations of growth factor (EGF, 10 pg/ml) induced low-frequency ERK activity pulses in a low percentage of cells (Albeck et al., 2013). The same biosensor, as well as a kinase translocation reporter (ERK-KTR (Regot et al., 2014)), showed that cells undergo spontaneous low-frequency ERK pulses when cultured in medium with 10% fetal bovine serum (Aoki et al., 2013; Aikin et al., 2020). Increasing the growth factor concentration or cell density resulted in more frequent ERK activity pulses and induced cell proliferation (Albeck et al., 2013; Aoki et al., 2013). Overexpressing EGFR or BRAF also induced high-frequency pulses and proliferation (Aikin et al., 2020). The pulse frequency-proliferation correlation has also been observed *in vivo* in mouse dermis and in intestinal organoids (Hiratsuka et al., 2015; Muta et al., 2018). This suggests that spontaneous, low-frequency ERK activation pulses are sufficient for single cell edge protrusion and migration, while higher frequency pulses direct cell proliferation.

Studies of oncogenic, constitutively-active mutants of the ERK pathway show that sustained ERK activity can induce collective cell migration. Imaging EKAR-EV in mouse embryonic fibroblasts (MEFs) expressing activating mutations in RAF and RAS induced sustained ERK activity by raising the baseline level of activity and lowering the probability for response to growth factor (Gillies et al., 2020). ERK signaling is rescaled by feedback loops so the peaks in activity induced by growth factors are attenuated (Gillies et al., 2020; Zhan et al., 2020). When introduced into RAS-less MEFs, oncogenic RAS enhances cell migration beyond that of wildtype RAS in random walk assays (Drosten et al., 2010), suggesting that an elevated baseline of ERK activity promotes migration. RAS oncogene-induced sustained signaling patterns were also observed in non-small cell lung cancer cell lines using optogenetic induction of SOS and ERK nuclear translocation as an indicator of ERK activation (Bugaj et al., 2018). Delayed inactivation kinetics resulted in sustained activity (Bugaj et al., 2018). Together, these studies indicate that sustained, elevated baseline ERK activity also promotes cell migration.

### Traveling waves of ERK activity in collective migration

Michiyuki Matsuda’s laboratory and others have now observed and dissected the ERK activity waves that travel through epithelial sheets during collective migration (Aoki et al., 2017; Aikin et al., 2020). EKAR-EV imaging of epithelial sheets in 10% serum showed that ERK activity increased in cells at the edge of a new wound and traveled into the migrating sheet of MDCK cells (Aoki et al., 2017). As the wave of ERK activity propagated into the sheet, cells experienced myosin II activation and migrated into the wound, moving in the opposite direction that ERK activity traveled (Aoki et al., 2017). ERK pathway stimulation by a light-inducible RAF (CRY2-BRAF, CIBN-EGFP-KRAS) or oncogenic BRAF^V600E^ also propagated waves of ERK, tracked by EKAR-EV or a kinase dead mCherry-ERK2 translocation reporter (Aoki et al., 2013; Hiratsuka et al., 2015; Aoki et al., 2017). The ERK activity wave required EGF signaling (Aoki et al., 2017). A complementary study imaged ERK-KTR in breast epithelial cell lines following induced expression of oncogenic, constitutively active RAF (BRAF^V600E^) and MEK (MEK2^DD^) (Aikin et al., 2020). The activating ERK pathway mutations again induced sustained ERK activity with small fluctuations around an elevated baseline (Aikin et al., 2020). Neighboring, uninduced MCF-10A cells exhibited collective migration towards the induced cells (Aikin et al., 2020). In a key proof-of-principle experiment a wave synthetically generated in the presence of an EGFR-ligand inhibitor by scanning light along a chain of MDCK cells expressing a photoactivatable BRAF system drove collective cell migration (Aoki et al., 2017). Thus, whether the initiating stimulus is the generation of a wound or direct ERK activation, ERK activity is propagated to neighboring cells and this wave of ERK activity is sufficient to induce collective cell migration towards the stimulus.

Further study of the ERK activity waves revealed that ERK signaling travels through epithelial sheets *via* EGF and mechanical signaling. ERK was found to activate A Disintegrin and Metalloprotease 17 (ADAM17), which triggered the shedding of membrane-bound EGF-family ligands as the ERK activity wave progressed through the epithelial sheet (Aoki et al., 2017; Aikin et al., 2020). ADAM17 and EGF ligand production were required for the propagation of ERK signaling waves in MDCK and MCF-10A cells (Aoki et al., 2017; Aikin et al., 2020; Lin et al., 2022) and *in vivo*, in mouse epidermis and intestinal organoids (Hiratsuka et al., 2015; Muta et al., 2018). Combinatorial knockout of the EGF ligands showed that at least one of the redundant EGF family ligands (EGF, HB-EGF, TGFα, or EREG) was required for the generation of the ERK activity waves (Lin et al., 2022). In the absence of EGF ligand production, EGF stimulation induced migration of leader cells only (Lin et al., 2022). Furthermore, propagation of the ERK activity wave required that neighboring cells have low ERK activity. Stimulation of the MCF-10A epithelial sheet with EGF ligand Amphiregulin blocked oncogene-induced migration of neighboring cells (Aoki et al., 2017) and bath application of EGF ligands failed to restore the waves in MDCK cells lacking expression of all four EGF ligands (Lin et al., 2022).

A study combining ERK biosensor imaging with traction force microscopy showed that mechanical signaling initiates and travels with the ERK activity wave (Hino et al., 2020). Cell stretch or extension preceded the ERK activity wave, while cell contraction and loss of traction force followed the ERK wave (Hino et al., 2020). Stretching a monolayer of MDCK cells activated EGFR, RAF, MEK, and ERK. Mechanical stress and tissue compression and stretch have similarly been shown to activate ERK in other systems, such as *Xenopus* embryos and cell monolayers (Hirata et al., 2015; Kinoshita et al., 2020). Photoactivation of ERK resulted in ROCK activation, cell contraction, and the propagation of stretch and ERK activation to the next layer of cells further into the sheet (Hino et al., 2020). Inhibition of ROCK, and therefore myosin II, blocked transmission of the ERK activity wave (Hino et al., 2020). These studies suggest that advancement of the leader cells toward free space exerts pulling forces on the adjacent follower cells, whose stretch activates ERK, which signals through ADAM17 for EGF release and ROCK for cell contraction. The ERK signal is relayed as EGF activates ERK in nearby cells and the contraction stretches adjacent cells further within the sheet (Hino et al., 2020). Thus, ERK signaling in leader cells sets off ADAM17/EGF and mechanical tension signals that travel through the sheet to induce collective migration.

### ERK and edge protrusion

EGF stimulation and ERK also drive the protrusion dynamics of leader cells and cells undergoing single cell migration (Mendoza et al., 2011b; Mendoza et al., 2015; Lin et al., 2022). Immunofluorescence showed that EGF stimulation induced phospho-ERK localization to the protruding edge of breast epithelial cells (Mendoza et al., 2011b). Consistent with this, activity of the EKAR-EV biosensor increased coincident with edge protrusion in MCF7 cells (Yang et al., 2018). Activity of the translocation reporter ERK-KTR lagged 6 min behind the peak in edge protrusion, as it must exit the nucleus to report ERK activity (Yang et al., 2018). We have found that both EGFR overexpression and BRAF^V600E^ expression, which increase the frequency and raise the baseline of ERK activity pulses respectively, promote lamellipodium protrusion (Mendoza et al., 2011b). This suggests that ERK activity fluctuations control edge motion. Indeed, it has been proposed that ERK functions in an excitable signaling network with RAS, phosphatidylinositol three- kinase (PI3K), and FAK signaling at the cell edge (Yang et al., 2018; Zhan et al., 2020). While ERK can promote protrusion, it also responds to local feed-forward and feedback signals (Zhan et al., 2020). Induction of protrusion *via* photo-activatable RAC, which induces actin polymerization, induced ERK translocation over the ensuing 30 min (Yang et al., 2018). The mechanism of this ERK activation is unknown, but could be due to stretch signaling observed in leader cells in collective migration, or *via* actin-mediated activation of RAS (van Haastert et al., 2017).

### ERK regulation of actin, adhesions, and myosin

Our finding that inhibition of MEK and ERK’s effector kinase p90 Ribosomal S6 Kinase (RSK) block edge protrusion within minutes supports a role for transcription-independent ERK signaling in cell migration and invasion (Matsubayashi et al., 2004; Mendoza et al., 2015; Samson et al., 2019). Indeed, ERK phosphorylates and controls the cytoplasmic machinery for each step of cell migration: protrusion, adhesion, and cell body contraction (Tanimura and Takeda, 2017). ERK directly regulates actin polymerization through phosphorylation and activation of the WRC (Mendoza et al., 2011b). Using computer vision to track the cell edge and actin polymerization and retrograde flow, we showed that ERK-induced actin polymerization converts initiated protrusions into prolonged events with the power to overcome membrane tension (Mendoza et al., 2015). ERK can also phosphorylate Cortactin, which promotes Cortactin’s activation of N-WASp (Martinez-Quiles et al., 2004; Kelley et al., 2010) for ARP2/3 activation in small 3D protrusions called invadopodia (Paterson and Courtneidge, 2018). Similar to the WAVE signal, Cortactin phosphorylation promotes lamellipodial persistence (Kelley et al., 2010).

ERK and RSK are activated in focal adhesions in response to integrin engagement (Renshaw et al., 1996; Schlaepfer et al., 1998; Fincham et al., 2000; Mendoza et al., 2011b). The scaffolding proteins Paxillin and GIT1 localize ERK to nascent adhesions (Ishibe et al., 2003; Yin et al., 2005; Vetterkind et al., 2013), while IQGAP1 localizes ERK to mature adhesions and bundled actin associated with myosin (Roy et al., 2005; Foroutannejad et al., 2014). ERK promotes adhesion disassembly for lamellipodium protrusion (Webb et al., 2004; Mendoza et al., 2011b). The mechanism of ERK-mediated adhesion disassembly is unknown, but likely involves ERK phosphorylation of FAK on a site that recruits a phosphatase that inactivates FAK (Hunger-Glaser et al., 2003; Zheng et al., 2009; Lavoie et al., 2020). ERK also controls regulators of RHO that promotes myosin II activity and contractility. ERK phosphorylates and activates GEF-H1, which induces RHO activation in response to mechanical force and cell spreading (Guilluy et al., 2011). RSK also phosphorylates and inactivates the myosin phosphatase regulatory subunit MYPT1 (Samson et al., 2019). This leads to increased myosin contractility for edge protrusion and retraction (Samson et al., 2019).

ERK also promotes cell migration through transcriptional induction of integrins, matrix metalloproteases, and the master regulator transcription factors for epithelial-to-mesenchymal transition (EMT) (Shin et al., 2010; Tsai and Yang, 2013; Lavoie et al., 2020). ERK phosphorylation of TCF transcription factors induces their expression of Immediate Early Genes, which go on to induce the expression of genes that promote cell migration. For example, Integrin β3 expression promotes adhesion signaling, while MMPs degrade barriers in 3D environments (McCawley et al., 1999; Woods et al., 2001; Wolf et al., 2013). Induction of EMT leads to the secondary expression of Integrins, MMPs, and intermediate filaments that promote the mesenchymal migration mode (Tsai and Yang, 2013; Wolf et al., 2013).

## Conclusions and future directions

**FIGURE 1:**
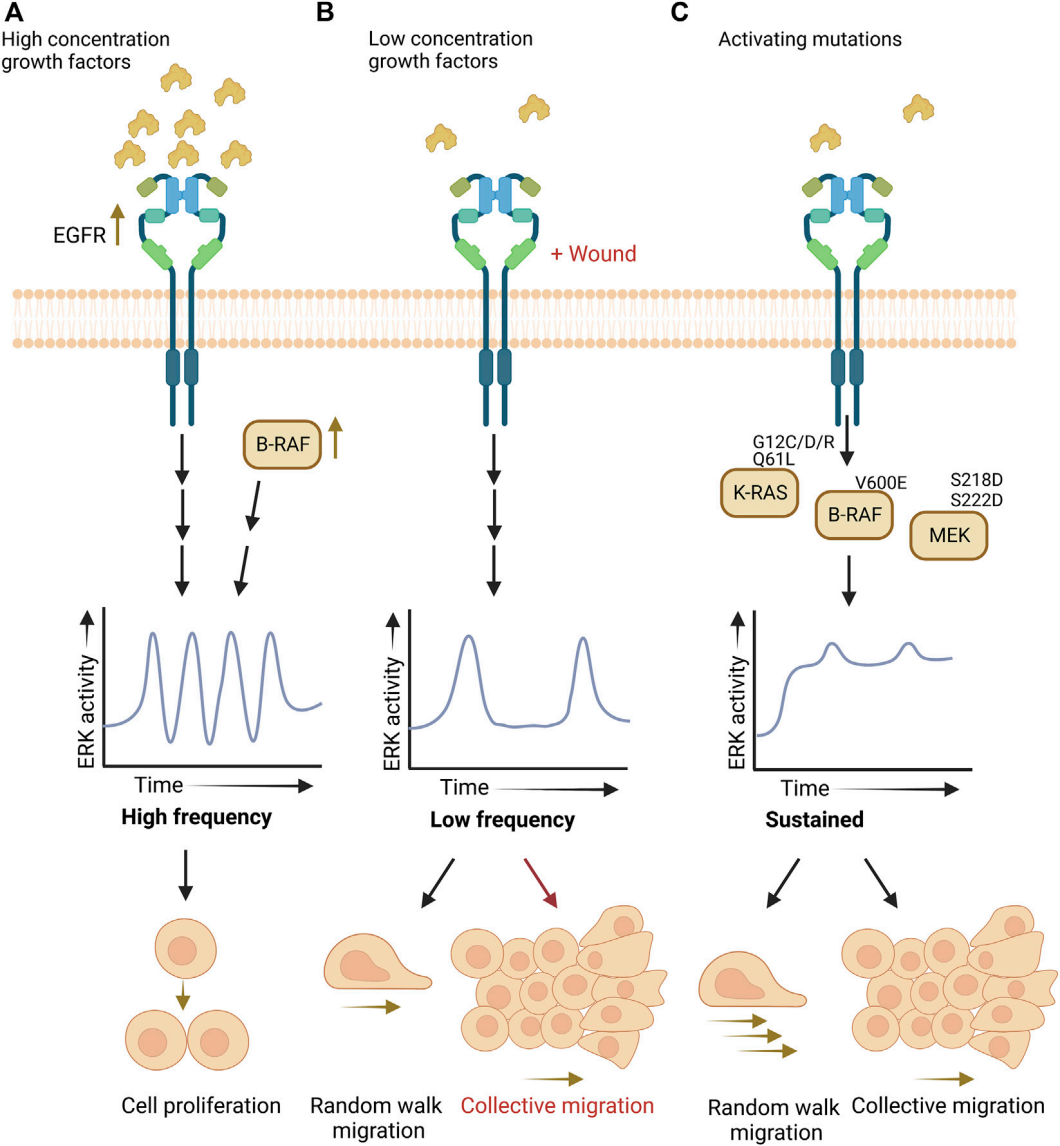
ERK activity patterns dictate cell proliferation versus migration. **(A).** High-frequency activity pulses promote proliferation. **(B).** Low-frequency pulses allow random walk migration, and collective migration when also stimulated by wounding. **(C)**. Activating mutations in RAS/RAF/MEK induce sustained ERK activity and promote random walk and collective migration.

Summarized in Figure 1, recent technological advances have uncovered low frequency ERK activity pulses in cells undergoing random walk migration and how sustained ERK activation induces cell contraction that creates waves of ERK activity for collective migration and invasion. Yet, key questions remain to be addressed on the timing and control of ERK signaling for cell movement during the different forms of physiological cell migration and invasion.

How pulses and an elevated baseline of ERK activity control random walk migration and initiate collective migration *in vivo* remains to be determined. At the highest growth factor and substratum stiffnesses, ERK activity peaks merge together, effectively raising the baseline of activity (Gillies et al., 2020; Farahani et al., 2021). Do sustained pulses dictate periods of movement in random walks? And do leader cells migrating up growth factor or stiffness gradients *in vivo* experience conditions that generate sustained signaling? Additionally, it remains to be determined how wound generation shifts the spontaneous ERK pulses to confer leader cell behavior. Experiments monitoring ERK activity in mouse skin found that the maximum amplitude of ERK activity decayed during propagation from random ERK activity pulses, but not from a wound (Hiratsuka et al., 2015). This was suggestive of ERK pulse synchronization, but whether wound generation shifts the baseline level of ERK is unknown. Lastly, as edge protrusion occurs on the order of tens of seconds (Ponti et al., 2004; Ji et al., 2008; Mendoza et al., 2015), understanding the timescale on which EKAR-EV and ERK-KTR sense ERK activity is needed before the meaning of correlations between sensor activity and edge motion can be deduced. As both EKAR-EV and ERK-KTR function as ERK substrates, new methods that directly monitor molecules of active ERK in space and time will be critical to answering these questions.
